# The audio-visual effects of rehabilitative landscapes on stress recovery in older adults: a case study of typical parks in Xi’an

**DOI:** 10.3389/fpsyg.2026.1874137

**Published:** 2026-07-24

**Authors:** Jinghua Dai, Xinlei Wang, Yongkai Chen, Yuji Qin, Yingtao Qi

**Affiliations:** School of Human Settlement and Civil Engineering, Xi’an Jiaotong University, Xi’an, China

**Keywords:** age-friendly landscape, audiovisual integration, landscape perception theory, stress recovery, therapeutic landscape

## Abstract

Based on the landscape perception theory and human factors methods, this study adopted eye-tracking and multimodal physiological monitoring to explore the effects of age-friendly rehabilitative landscapes on stress recovery in older adults. A total of 44 healthy older adults were recruited. Fourteen landscape scenes from five types of parks in Xi’an were selected to collect physiological data under visual-only and audio-visual combined conditions. The results showed three key findings. First, the main effect of visual environment was significant (*p* < 0.05). Landscapes with moderate plant diversity and static water features produced the optimal stress recovery effects. Second, the audio-visual interaction effect was significant (*p* < 0.05). Auditory stimuli did not universally promote stress recovery, and their effects were regulated by visual landscape types. Third, eye movement data indicated excessive audio-visual stimuli might reduce recovery efficiency. Open spaces and static water features showed strong anti-interference capabilities. Sound cues effectively drew older adults’ visual attention to water landscapes. This study suggests prioritizing landscapes with moderate plant complexity and static water features. The sound volume of dynamic water scenes should be controlled, and open spaces can be designed to reduce cognitive load. The findings deepen the understanding of how audio-visual landscapes affect stress recovery in older adults, and provide empirical evidence for the design and optimization of age-friendly landscapes in urban parks and community green spaces.

## 1 Introduction

### 1.1 Rehabilitative landscapes in response to aging needs

Population aging stands as one of the most prominent global demographic trends (Liu and Ye, 2024). In China, the proportion of older adults keeps rising due to longer life expectancy and declining birth rates. Aging is often accompanied by deteriorating physical functions, increased psychological stress and reduced quality of life. These changes create greater demands for health-supporting outdoor spaces (Li and Feng, 2026). Against this backdrop, rehabilitative landscapes have gained wide attention for their capacity to boost physical activity, relieve mental stress and improve overall well-being. Exploring evidence-based design strategies for rehabilitative landscapes has become a key research topic in landscape architecture and public health.

Existing studies have proven that natural environments help alleviate stress and improve mental health (Ning and Yang, 2024). Nevertheless, most current research focuses solely on visual landscape features. The combined effects of visual and auditory stimuli remain understudied, particularly for older adults. Meanwhile, objective physiological evidence supporting the restorative functions of therapeutic landscapes is still insufficient (Cai, 2003). These research gaps hinder the formulation of evidence-based design guidelines for age-friendly therapeutic landscapes.

### 1.2 Landsenses theory for the development of green elderly care

Landsense refers to landscape perception. As the perceiver in landscape spaces, humans receive external environmental stimuli. These stimuli trigger complex psychological and physiological changes, and further guide corresponding behaviors (Shu et al., 2019). It reflects the interactive relationship between people and landscape environments. Landsenses theory emphasizes the systematic and dynamic nature of human perception. It places humans at the core of research and explores how environments regulate mental states through multiple sensory pathways, including vision, hearing and smell. This theory offers a crucial perspective for explaining how rehabilitative landscapes work. A landscape is not merely a static physical space. Instead, it acts as a dynamic medium to facilitate physical and mental recovery via integrated sensory experiences (Sun and Allen, 2024).

Rehabilitative landscape is an interdisciplinary field combining landscape design and rehabilitation medicine (Li et al., 2018). In the narrow sense, it refers to therapeutic landscapes affiliated with medical institutions. In the broad sense, it covers all landscape spaces with health benefits. Humans possess an innate biophilia, which drives people to seek natural environments for energy restoration (Wilson, 1984). Therefore, well-designed age-friendly rehabilitative landscapes can positively improve older adults’ physical conditions and social interactions (Wang, 2022).

Nowadays, green elderly care has gained increasing attention. It advocates improving older adults’ quality of life via natural environments and eco-friendly designs. In the application of landsenses theory, a landscape serves as a perceptual interface connecting humans and the surroundings. Optimizing environmental perception helps older adults regulate emotions and relieve stress, and encourages their social participation. Older adults commonly experience sensory decline and cognitive changes. Guided by landsenses theory, rehabilitative landscape design can respond to their special perceptual needs. Through coordinated spatial planning and environmental configuration, designers can enhance environmental readability, safety and comfort. These improvements strengthen older adults’ sense of control and belonging, and promote their spontaneous physical and mental recovery (Millward and Appleton, 1988). This study adopts landsenses theory to systematically explore how multi-sensory integration in rehabilitative landscapes affects older adults’ stress recovery. It provides empirical evidence and design guidelines for the practice of green elderly care.

### 1.3 Research status at home and abroad

China is currently experiencing severe population aging. Mental stress among older adults has become prominent. Low-cost natural healing landscapes have emerged as an important non-pharmaceutical intervention method. Nevertheless, literature review reveals obvious research gaps in empirical studies on audio-visual therapeutic landscapes for the elderly. This section summarizes relevant research progress at home and abroad.

Extensive studies have been conducted on rehabilitative landscapes, landsenses theory and stress recovery. These findings lay solid theoretical and methodological foundations for the present research.

Research on rehabilitative landscapes started earlier overseas and has formed a systematic theoretical framework. In 1983, Ulrich proposed the stress reduction theory. He argued that landscapes containing three types of affective elements can relieve psychological and physiological stress responses (Ulrich, 1983). Kaplan and colleagues found that outdoor landscapes with natural elements can effectively reduce anxiety and depression, and improve mood and cognitive function among older adults in senior care facilities (Kaplan and Kaplan, 1983). Subsequent research expanded the scope of rehabilitative landscapes from medical facilities to public green spaces. Scholars have also shifted research focus from the general public to older populations, exploring safety, accessibility and social support in age-friendly design.

Although domestic research started relatively late, it has developed rapidly. Chinese scholars have explored classification systems, design principles and localized practices of rehabilitative landscapes. Virtual reality and physiological monitoring technologies have also been adopted for empirical research, providing preliminary references for age-friendly landscape applications (Shu et al., 2019).

Studies on landsenses theory have evolved from single visual evaluation to multi-sensory integrated analysis. In the mid-20th century, American scholar Litton R. Burton took urban forest landscapes as research objects. He established a landscape evaluation framework consisting of six factors and seven categories. His work realized the structured classification and description of forest landscape features from the perspective of natural resources and ecosystem services, laying a methodological foundation for subsequent landscape perception research (Litton, 1968). On this basis, Lang Jon T. analyzed the correlation between landscape features and human behaviors from the perspective of environmental psychology. He pointed out that landscape charm is the core driver of human landscape perception and behavioral activities (Lang, 1974). With the development of environmental psychology and neuroscience, relevant research has extended to auditory, olfactory and other sensory dimensions, as well as perceptual interactions and cultural differences.

Chinese researchers have introduced overseas theories and carried out localized explorations. Liu Binji and his team analyzed how landscape elements affect human psychological responses from the perspectives of visual evaluation and environmental perception. They stated that landscape perception research should serve ecological protection and the improvement of human living environments (Liu, 2022). The introduction of landscape urbanism has further broadened the research scope. It regards landscapes as “spatial syntax” and emphasizes their systematic function in connecting spaces and conveying meanings (Yang, 2012). The application of internet, artificial intelligence and multimodal physiological monitoring has innovated data collection methods. For example, recent studies have adopted image recognition and generative adversarial networks (GANs) to develop behavior-driven spatial generation methods. These techniques realize the bidirectional prediction of space and human behaviors, offering new approaches to analyze complex spatial activities. The combination with emerging theories and technologies also expands the application potential of landsenses theory in analyzing dynamic human behaviors (Jin et al., 2026).

Overseas scholars have established mature evaluation systems for restorative environments. The Perceived Restorativeness Scale (PRS) is widely used. A large number of experiments have verified that natural scenes and sounds such as running water, bird songs and wind can effectively relieve stress and promote mental recovery (Yang and Kang, 2005).

Domestic research has also made rapid progress in this field. Scholars have adopted eye-tracking, electroencephalography and heart rate variability indicators to explore environmental effects on stress recovery. However, most existing studies target young people. Empirical analysis on stress recovery mechanisms of older adults under audio-visual combined environments is still insufficient.

Overall, current research has achieved fruitful outcomes in the theoretical construction of rehabilitative landscapes, exploration of landsenses mechanisms and empirical verification of stress recovery. Three main limitations still exist. First, systematic studies focusing on older adults are inadequate, especially researches on multi-sensory interaction mechanisms. Second, most studies fail to fully combine subjective evaluation with objective monitoring, making it hard to reveal the internal rules of environmental perception. Third, the interdisciplinary integration of landsenses theory, human factors and rehabilitative landscape design needs to be strengthened.

Against the above research gaps, this study selects older adults as participants. It combines landsenses theory and human factors methods, and integrates subjective and objective data. This paper explores the effects of rehabilitative landscapes on older adults’ stress recovery under audio-visual conditions, so as to provide scientific references for the optimization of age-friendly landscapes (see Table 1). The above unresolved research problems are the core starting point of this empirical study conducted in urban parks of Xi’an.

**Table 1:** Comparison of domestic and overseas studies on audio-visual perception of restorative landscapes and summary of research gaps addressed in this work.

Dimension	Overseas research	Domestic research	Research gaps filled by this study
Research subjects	Elderly adults are the core research participants	Most studies recruit young participants; specialized experiments targeting the elderly are scarce	Conduct audio-visual landscape experiments focusing on healthy elderly people
Measurement methods	Combined multimodal physiological monitoring (EEG, eye-tracking, skin conductance) and subjective questionnaires have become standard practice	Questionnaires dominate existing research; multimodal physiological measurement is rarely adopted	Integrate Kano model subjective questionnaire, four physiological indicators and eye-tracking for joint evaluation
Sensory research perspective	Systematic and mature studies on individual and interactive effects of multi-sensory stimuli (vision, audition, etc.)	Most studies only focus on single visual landscapes	Explore the synergistic effects of integrated audio-visual stimuli on stress recovery

### 1.4 Research objectives

From the perspectives of restorative environment research and multi-sensory perception, combined with the development demands of healthy aging, this study systematically explores the impacts of age-friendly rehabilitative landscapes on older adults’ stress recovery. Four specific research objectives are proposed as follows:

(1) To clarify older adults’ subjective needs and landscape preferences. This study adopts questionnaires based on the Kano model to identify older adults’ demands for landscape safety, comfort, natural elements and supporting facilities. The findings can provide a reference for the construction of age-friendly rehabilitative landscapes.
(2) To analyze the effects of different landscape elements on older adults’ stress recovery. Multimodal physiological monitoring technologies including skin conductance, heart rate variability, electroencephalography and eye-tracking are applied to examine the restorative effects of space types, plant configurations, water features and landscape structures.
(3) To compare the differences in stress recovery between visual-only stimuli and audio-visual combined stimuli. Based on multi-sensory perception mechanisms, this paper discusses whether audio-visual integration can enhance restorative effects, and analyzes the interaction between visual and auditory environments.
(4) To put forward empirical evidence and optimization strategies for age-friendly rehabilitative landscape design. By integrating older adults’ subjective demands and objective physiological responses, this study proposes design suggestions for rehabilitative landscapes oriented to healthy aging, and provides scientific references for the renewal of age-friendly urban parks.

## 2 Methods and experimental procedures

To fully explore older adults’ perceptual demands for rehabilitative landscapes, this study conducted questionnaire surveys based on the Kano model from April to June 2024 at five typical parks in Xi’an. A total of 212 valid questionnaires were collected. The survey results guided the selection of experimental scenes and landscape elements, and provided references for the subsequent physiological experiments. On this basis, we carried out further physiological experiments according to the actual features of park environments.

### 2.1 Selection of restorative environments

A restorative environment refers to a setting that helps people recover from mental fatigue and negative emotions and physiological responses caused by stress (Su and Xin, 2010). This study divides restorative environments into visual and auditory categories. According to Landscape Design Elements (Booth et al., 1989), core landscape components include terrain, plants, buildings, pavements, landscape structures and water bodies.

Buildings mainly serve indoor spaces and exert limited effects on older adults’ physical and mental recovery. Pavements focus on functional attributes such as anti-slip performance, durability and accessibility. Since this experiment adopted video stimuli instead of on-site tactile tests, buildings and pavements were excluded from experimental variables. Finally, we defined experimental variables from four dimensions: space, plants, water features and landscape structures, forming 13 experimental scenes in total (see Table 2).

**Table 2:** Restorative environment. A, B, C, and D correspond to four landscape categories: space, vegetation, water features and built structures; A1–D3 and C4 are the codes of the 13 experimental landscape scenes.

Restorative environment	Environmental classification			
A. Spatial type	A1 private space	A2 Semi-private/SEMI-open space	A3 open space	
B. plant species richness	B1 high plant species richness	B2 medium plant species richness	B3 low plant species richness	
C. water feature type	C1 static water feature	C2 flowing Water	C3 fountain	C4 waterfall
D. structure type	D1 leisure-oriented structure	D2 traffic-oriented structure	D3 art-oriented structure	

To quantify plant species richness, the Shannon Diversity Index (H) was adopted to reflect the richness and evenness of plant communities in each experimental scene (Ye et al., 2023). The specific calculation equation is presented below:


$$H=-\sum [P_{i}*\log_{2}P_{i}]$$

Where:

Pi = the proportion of species i in the total sample, i.e., the probability of species i appearing in the total sample;

H = Shannon Diversity Index.

### 2.2 Collection and processing of experimental materials

Five typical urban parks in Xi’an were selected as research sites to collect representative rehabilitative landscape scenes. Thirteen landscape scenes were screened out based on landscape characteristics, usage frequency by older adults and spatial accessibility. These scenes were divided into four categories. Specifically, A1, A2 and A3 came from City Wall Park; B1, B2, and B3 from Fengqing Park; C1, C2, C3, and C4 from Lianhu Park; D1, D2, and D3 from Changle Park. All scenes were used as visual stimuli in subsequent experiments.

The five selected parks, namely City Wall Park, Xingqing Palace Park, Fengqing Park, Lianhu Park, and Changle Park, cover diverse locations, user groups and spatial structures (see Table 3). The samples are highly representative and can support the comparative experiments on how different landscape elements affect older adults’ stress recovery.

**Table 3:** Research location.

NO.	Park name	Area (hectares)	Type	Level
1	Huancheng Park	2,400	Linear Park	Municipal
2	Xingqinggong Park	48	Comprehensive Park	Municipal
3	Fengqing Park	25	Comprehensive Park	District
4	Lianhu Park	7	Historical Park	Residential
5	Changle Park	17	Community Park	Residential

Each of the four major landscape categories contains multiple sub-scenes, including 3 scenes for Category A, 3 for Category B, 4 for Category C and 3 for Category B. No single video was used to stand for an entire landscape type. Multiple repeated samples within the same category can eliminate the interference caused by unique features of individual sites, such as trees, landscape ornaments and terrain. Therefore, the experimental results can reliably reflect the general restorative effects of each landscape category.

Preliminary field surveys and trial video shooting were conducted at the five selected parks from July 2 to 6, 2024, to identify sites with typical restorative environmental features. Formal video and audio recording was carried out on July 10 and 11.

A Sony Alpha 7C camera was used to capture 1-min fixed-point videos at each site, while a ZOOM H5 digital recorder simultaneously recorded on-site ambient sounds. All raw video and audio footage was edited with Adobe Premiere Pro 2023. In total, 13 silent video clips and 13 audio-equipped video clips were produced, with each clip lasting 30 s for the perception experiment on older adults’ stress recovery. The corresponding video scenes of each restorative environment are presented in Table 4.

**Table 4:** Restorative environment corresponding to video scenarios.

Torative environment	Video scenarios			
A. Spatial type	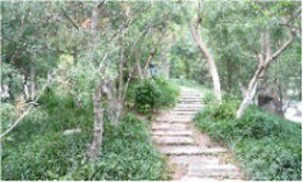 A1 private space	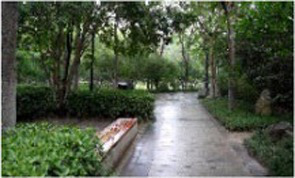 A2 semi-private/semi-open space	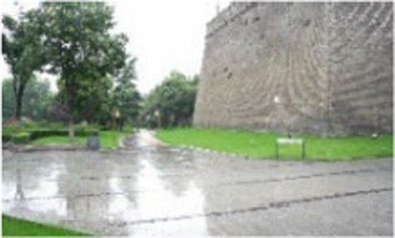 A3 open space	
B. Plant species richness	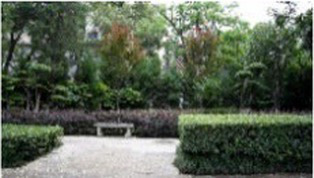 B1 high plant species richness (*H* = 1.91)	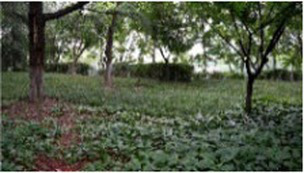 B2 medium plant species richness (*H* = 1.07)	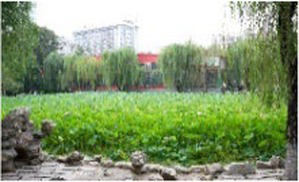 B3 low plant species richness (*H* = 0.15)	
C. Water feature type	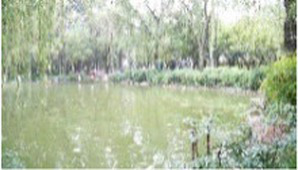 C1 static water feature	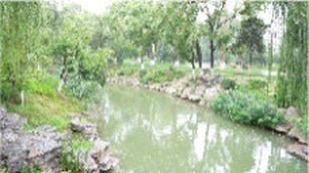 C2 flowing water	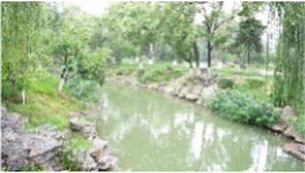 C3 fountain	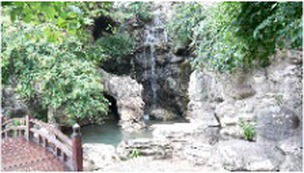 C4 waterfall
D. Structure type	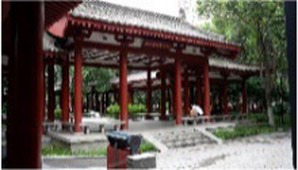 D1 leisure-oriented Structure	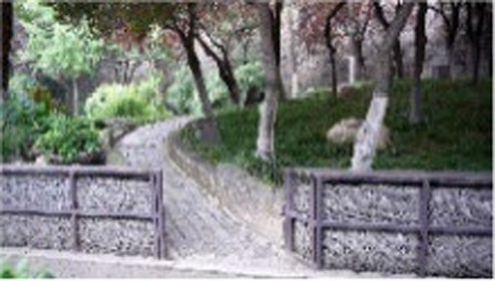 D2 traffic-oriented Structure	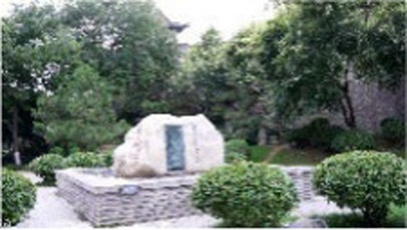 D3 art-oriented Structure	

### 2.3 Standardization of experimental stimuli

All videos were captured with identical equipment and settings to eliminate confounding variables. A fixed camera position was adopted for shooting. The camera height was set to match the eye level of a standing adult. Participants watched the videos at a distance of 60–80 centimeters, and the lens was kept horizontal to ensure a consistent viewing perspective across all stimuli.

Shooting was arranged under stable weather conditions with no rainfall or extreme weather. All footage was recorded during similar time periods to maintain uniform natural light. We also avoided crowds, passing vehicles and other unexpected disturbances during filming.

Each scene was recorded for 1 min on a 24-inch monitor, with on-site sounds captured simultaneously. Adobe Premiere Pro 2023 was used for post-editing. We selected continuous 30-s segments with stable images and sounds free from external interference as the final stimuli. All videos were exported with the same resolution, duration and file format.

The auditory stimuli consisted of original on-site sounds, including bird songs, wind, running water and general ambient noise in parks. This study focused on the overall effects of audio-visual environments versus visual-only environments on older adults’ stress recovery, rather than the independent effects of individual sound types. Therefore, environmental sounds were treated as an integral part of landscape experience without further classification.

For the audio-visual group, original natural sounds were retained without background music or artificial sound effects. The volume of all audio clips was calibrated to the same level to ensure equal loudness. The visual-only group used the exact same video content but with all sounds removed. The two groups differed only in auditory conditions.

Throughout the experiment, all participants used the same display device and maintained a fixed viewing distance and sitting posture. The brightness, contrast and volume of the display remained unchanged for all trials. These measures strictly controlled irrelevant interfering factors.

### 2.4 Selection of experimental indicators

Skin Conductance Level (SCL) is a typical indicator for assessing emotion, stress and autonomic nerve activity. Its value rises when participants feel tense and falls when they enter a relaxed state (Li et al., 2010). Heart Rate Variability (HRV) reflects the balance between the sympathetic and parasympathetic nerves. It serves as a key parameter to evaluate cardiac autonomic regulation, psychological stress and neural tension. Frequency-domain analysis of HRV is commonly applied, and the LF/HF ratio is used to judge individual stress levels. A higher LF/HF ratio indicates greater stress or anxiety, while a lower value represents a more relaxed state.

Electroencephalogram (EEG) records electrical activities of the cerebral cortex via scalp electrodes. It contains multiple frequency bands. Among them, alpha waves (8–12 Hz) and beta waves (12–30 Hz) are widely adopted in perceptual experiments (Zhang et al., 2023). The *α*/*β* ratio of EEG signals is selected as the physiological indicator in this study. A higher α/β value generally means participants are more relaxed, and a lower value corresponds to a tense state (Yang et al., 2020).

In eye movement research, visual response indicators can effectively evaluate individuals’ physical and psychological reactions in specific scenarios. They help analyze attention distribution, cognitive load, emotional responses and visual exploration patterns. An enlarged pupil diameter usually suggests intense emotions or high cognitive load. Increased blink frequency is closely associated with tension, anxiety and fatigue. Saccade refers to rapid eye movements between different fixation points. A higher saccade frequency means participants are engaged in complex visual tasks with elevated cognitive load. This study adopts pupil diameter, blink frequency and saccade frequency as three core eye movement indicators. In general, higher values of these three indicators reflect greater cognitive load and psychological tension. We use them to explore older adults’ visual preferences and attention characteristics toward rehabilitative landscapes.

### 2.5 Selection of experimental equipment

This experiment adopted the ErgoLAB V3 human-machine-environment synchronous platform for simultaneous data collection. The collected data included neural signals (EDA and HRV), brain cognitive data (EEG system) and visual data (eye-tracking system).

All experimental devices were provided by the research support program of Kingfar International Inc. Detailed equipment information is shown in Table 5.

**Table 5:** Detailed information of experimental equipment.

Tablemeasured indicators	Equipment name	Collected data	Equipment list
Skin electrodermal signal	ErgoLAB EDA Wireless EDA Sensor	SCL (Skin Conductance Level)	BIO sensor, finger clip, charging cable
Heart rate variability (HRV)	ErgoLAB Chest Biosensing Smart Wearable Chest Strap Sensor	LF/HF power ratio	Modular chest strap sensor, binding band, charging cable
EEG data	BitBrain EEG 16-channel Semi-dry Electrode Wearable EEG Device	Brain waves	16-channel EEG cables, EEG cap, EEG amplifier, back strap, EEG charger
Eye-tracking data	Tobii Pro Fusion Non-contact Mobile Terminal Eye Tracker	Fixation behavior and visual responses	Tobii Pro Fusion remote eye tracker, charging cable, behavioral experiment camera

### 2.6 Experimental design

**Figure 1:**
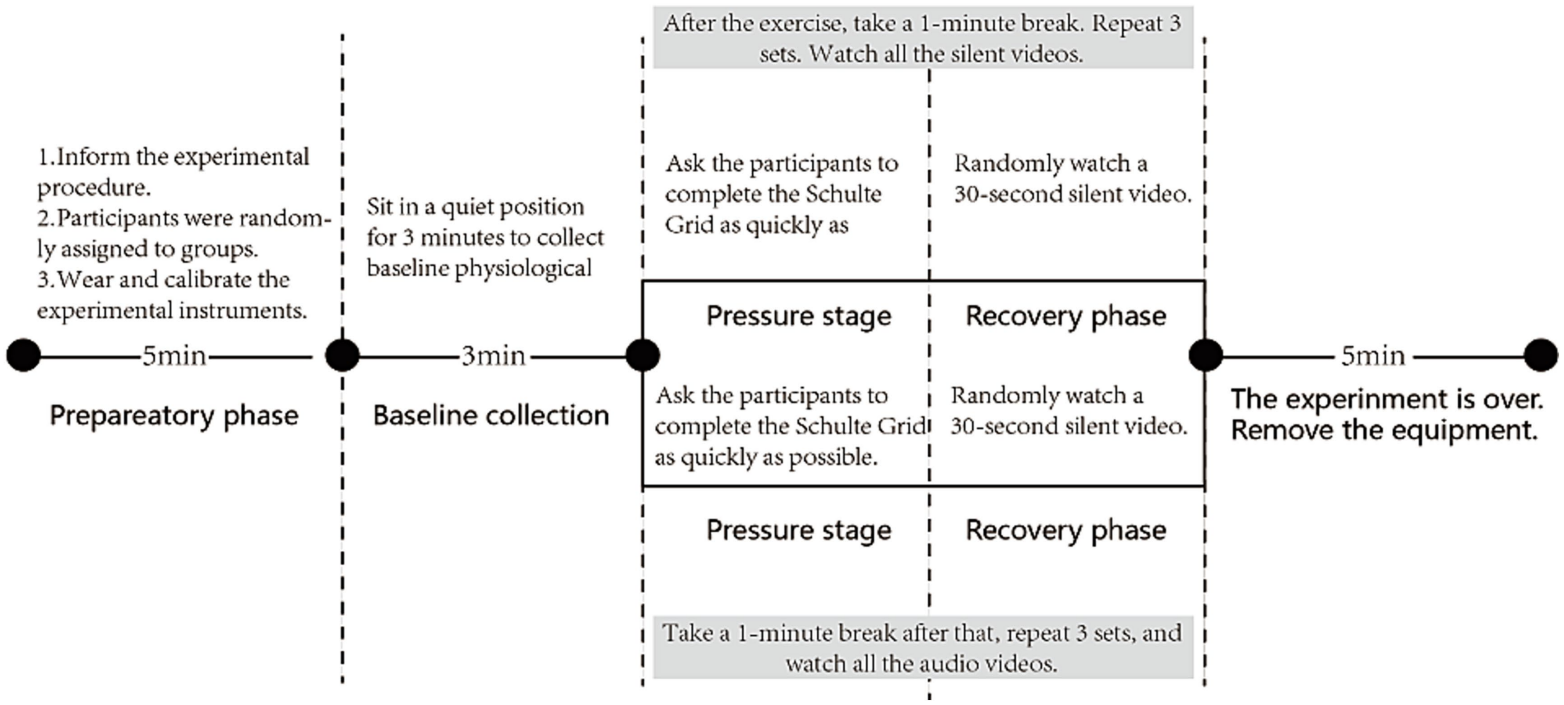
Experimental process.

Physiological indicators were recorded when participants watched videos of restorative environments under audio-present and audio-absent conditions. Participants were randomly assigned to two groups. The two groups followed identical experimental procedures, with the only difference being the audio setting of video stimuli. The overall experimental workflow is illustrated in Figure 1.

The experiment consisted of several sequential steps. First was the preparation phase. Researchers explained the procedures to participants, assigned them to groups randomly, and helped them put on and adjust experimental devices. Next came a 3-min baseline recording session, during which participants sat quietly to collect physiological data at rest. Then the core experimental phase began. Before each video clip, participants completed a 30-s Schulte Grid task to induce psychological stress. After the stress task, participants watched a 30-s video. One group viewed videos with original sounds, while the other watched silent videos. There was a 1-min rest interval after each trial. This process was repeated 13 times for all landscape scenes. Finally, all devices were removed, and participants were dismissed.

Given that all participants were older adults, we used a fixed playback order for all stimuli. This avoided extra cognitive load caused by random sequence and ensured reliable participation. All participants viewed the 13 scenes in the same order, so the background conditions remained consistent across trials.

The Schulte Grid task was applied before each landscape stimulus to mitigate sequence effects, fatigue and aftereffects of previous stimuli. It standardized participants’ stress levels for every trial. A 1-min break was arranged after each round to reduce cumulative fatigue from continuous stimulation. The total duration of the experiment was controlled within the endurance range of older adults. Researchers monitored participants throughout the experiment. The test would be terminated immediately if anyone felt fatigued or unwell. Figure 1 demonstrates the complete timeline covering baseline recording, stress induction and landscape viewing.

The Schulte Grid task was used prior to each landscape viewing session to ensure participants started with similar stress levels for all stimuli. As a classic visual search and attention test, this task requires participants to find randomly arranged numbers in sequence within a limited time. It demands sustained focus, rapid information processing and visual searching, which effectively raises cognitive load and induces psychological stress. For this reason, it is widely adopted in studies on cognitive function and stress recovery.

In formal trials, each participant completed a 30-s 5 × 5 Schulte Grid test before viewing landscape videos. Physiological signals were collected synchronously during video playback to evaluate the restorative effects of different landscapes (Yang and Kang, 2005). The Schulte Grid served as a standardized cognitive stressor. Changes in SCL, LF/HF and EEG readings were then measured to reflect variations in stress and recovery status.

This study focused on the immediate stress recovery responses of older adults rather than long-term recovery effects. Extended visual stimuli would easily cause visual fatigue, reduced attention and cumulative effects across trials, which would compromise result reliability. Therefore, each video clip was set to 30 s. This duration allowed participants to fully perceive landscape information while avoiding experimental biases.

To guarantee consistency of experimental materials, all scenes were recorded for 1 min with synchronized audio. We selected continuous 30-s segments as formal stimuli. These segments featured stable images, even lighting and no obvious human or vehicle interference. All videos shared the same shooting height, focal length, duration and playback mode to minimize confounding variables.

This research adopted the physiological change rate (rather than absolute values from the recovery phase) for data analysis. This method reduces the impacts of individual physiological differences and short sampling duration on experimental results.

### 2.7 Participants

**Figure 2:**
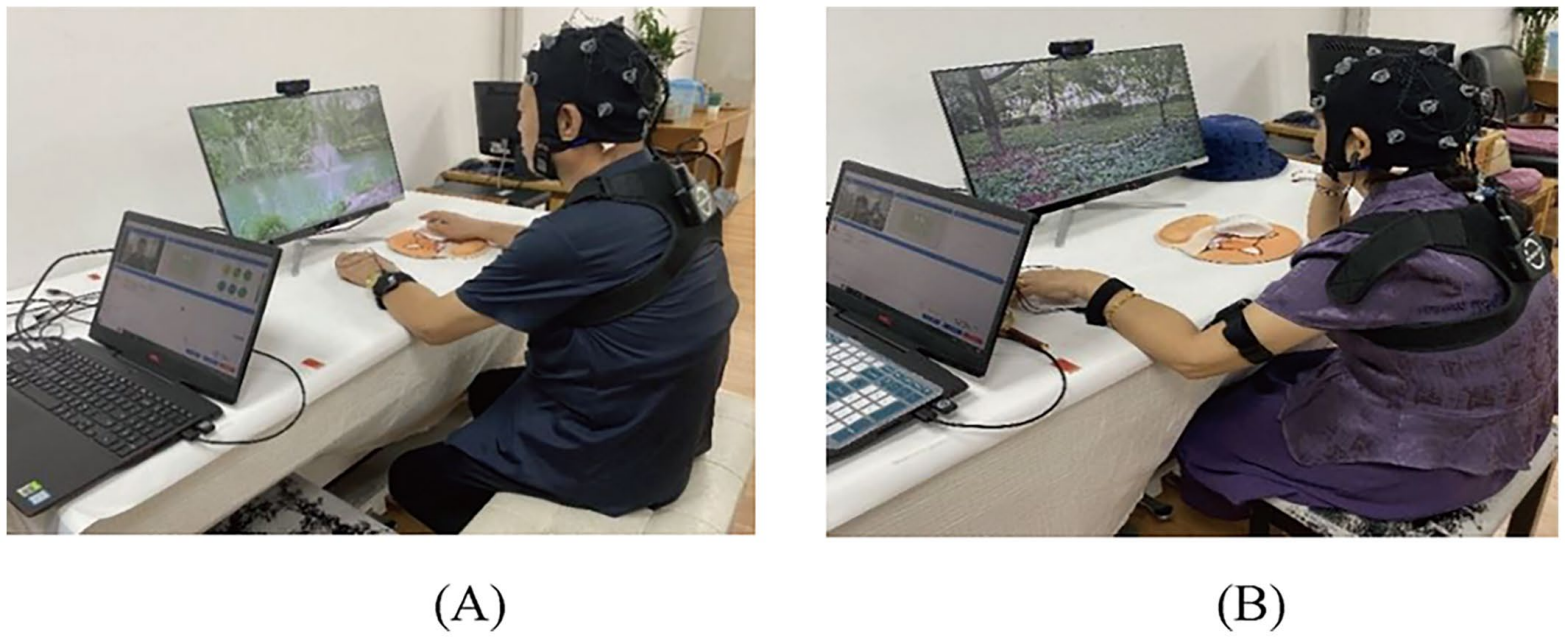
Experimental environment and experimental process. **(A)** Test scene of an elderly male participant. **(B)** Test scene of an elderly female participant.

A total of 44 volunteer participants aged 60 years or older were recruited for this study. They were randomly divided into the visual-only group and the audio-visual group, with 22 participants in each group (see Figure 2). All participants were capable of completing the experiment independently and signed informed consent prior to the test.

Strict inclusion criteria were formulated to ensure reliable and consistent experimental data:

(1) Aged 60 years or above;
(2) Having normal or corrected vision to clearly identify visual stimuli;
(3) Possessing normal hearing to perceive audio stimuli;
(4) Free from physical and mental illnesses, and able to finish experimental tasks independently;
(5) Having no severe ocular or neurological diseases that may interfere with visual perception and physiological signal collection.

To avoid interference with physiological indicators, all participants were required to refrain from caffeine, alcohol, drugs and other stimulating substances within 24 h before the experiment. They were also asked to maintain regular routines and avoid strenuous exercise and excessive fatigue.

This study primarily recruited community-dwelling older adults with independent mobility to guarantee smooth participation. If any participant felt unwell or could not cooperate during the experiment, the test would be terminated immediately and the relevant data would be excluded.

## 3 Data analysis and experimental results

ErgoLAB 3.0 software was used for the preprocessing of collected physiological data. Skin conductance, heart rate variability and electroencephalogram readings are susceptible to skin conditions and individual physiological differences. To eliminate such interference, the physiological response change rate (R) was adopted for data processing (Packard and Boardman, 1999).

The calculation equation is defined as follows:


$$R=\frac{F1-F2}{F1-F0}$$

Where:

F1 = Mean value of physiological indicators during the stress induction phase.

F2 = Mean value of physiological indicators during the video viewing (recovery) phase.

F0 = Mean value of physiological indicators at the resting baseline.

A new Schulte Grid task was administered before each landscape stimulus. The values of F0, F1 and F2 were calculated independently for every scene, and the corresponding stress recovery rate was computed accordingly. This approach eliminated the residual effects of previous trials. Before the formal experiment, all participants completed the unified stress induction task. Real-time monitoring verified obvious changes in their physiological indicators compared with the resting state. Thus, the value of $F_{1}-F_{2}$ never approached zero in valid datasets. During data preprocessing, outliers and data drift caused by equipment interference were removed to guarantee stable and reliable calculations of the recovery rate.

A higher value of R indicates a better restorative effect of the landscape scene. Unless otherwise specified, the physiological change rate was adopted for the analysis of skin conductance, heart rate variability and electroencephalogram data.

For eye movement data, we calibrated coordinates to ensure accurate mapping between eye tracking records and the display screen. Device accuracy was checked via pre-experiment calibration, and recalibration or offset correction was conducted when necessary. Samples with missing data or low acquisition rates were excluded before visual analysis.

**Figure 3:**
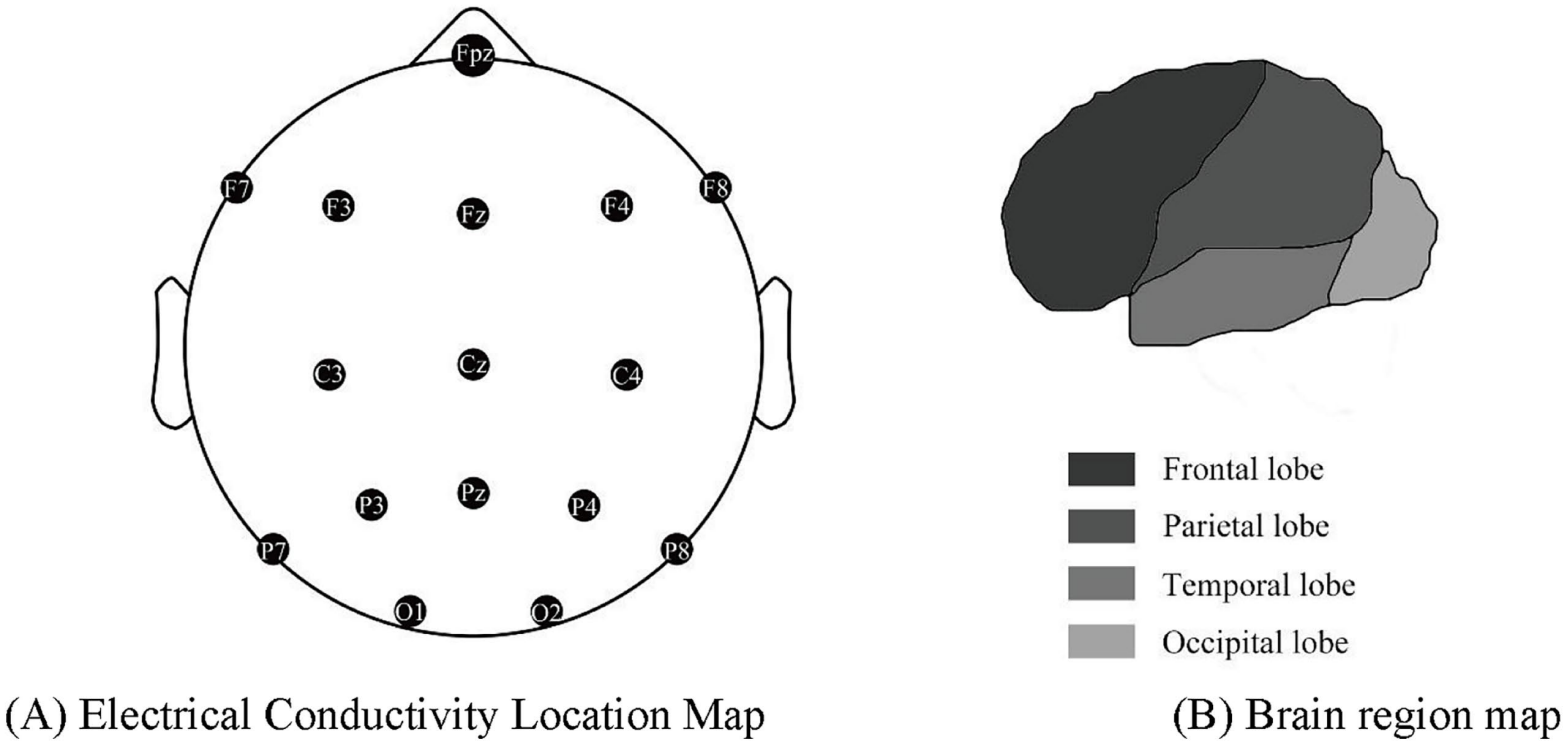
The location of the conductance channels and the brain region. **(A)** Distribution map of 16 EEG electrodes placed following the international 10-20 system, covering frontal, central, parietal, temporal and occipital lobes.**(B)** Schematic diagram of brain functional regions, including frontal lobe, parietal lobe, temporal lobe and occipital lobe, matching the electrode positions for regional EEG analysis.

EEG data were processed using the EEGLAB plugin in MATLAB. The procedures included electrode positioning, filtering, re-referencing and artifact removal, as well as independent component analysis (ICA). The LETSWAVE plugin was then used for data segmentation and Fourier transform to extract EEG signals across different time periods. Six electrode channels were selected for analysis (Kacha et al., 2015), including F3 and F4 in the frontal lobe for language and emotional cognition, P7 and P8 in the parietal lobe for sensory processing, and O1 and O2 in the occipital lobe for visual and object recognition (see Figure 3).

To verify the effectiveness of the Schulte Grid stress induction task, we compared the mean values of SCL, LF/HF and *α*/*β* between the resting baseline and post-stress phases. Across all landscape scenes and two audio conditions, participants presented typical stress responses: increased SCL and LF/HF values, and decreased α/β values. The results confirmed that the Schulte Grid task could stably and effectively induce psychological and physiological stress, proving the reliability of the stress induction procedure (Table 6).

**Table 6:** Comparison of mean physiological indicators between baseline rest stage and stress stimulation stage with Schulte Grid task.

Scene ID	Stage	SCL Audio	SCL Silent	LF/HF Audio	LF/HF Silent	α/β Audio	α/β Silent
	Mean value at baseline rest	2.36	2.16	1.51	3.03	11.33	8.33
A1	Value after stress stimulation	3.02	3.58	2.30	5.33	11.00	1.30
A2	Value after stress stimulation	3.08	3.53	2.26	5.78	0.78	2.56
A3	Value after stress stimulation	3.12	3.53	1.89	3.24	8.75	7.00
B1	Value after stress stimulation	3.08	3.74	2.65	3.31	8.50	0.94
B2	Value after stress stimulation	2.99	3.42	2.20	3.17	5.57	1.90
B3	Value after stress stimulation	3.05	3.56	2.11	3.09	7.25	4.20
C1	Value after stress stimulation	3.06	3.65	1.96	3.51	6.40	1.20
C2	Value after stress stimulation	3.09	3.62	2.06	3.37	8.50	0.76
C3	Value after stress stimulation	3.02	3.58	1.89	3.85	7.20	1.14
C4	Value after stress stimulation	3.21	3.58	1.90	3.54	10.00	5.20
D1	Value after stress stimulation	3.06	3.46	3.63	3.15	8.25	1.50
D2	Value after stress stimulation	3.20	3.61	3.72	5.37	6.33	0.80
D3	Value after stress stimulation	3.05	3.74	3.03	4.54	8.75	1.00

All data were analyzed using the SPSSAU online statistical platform. A two-way analysis of variance (ANOVA) was first conducted to examine the main effects of auditory environments, visual environments, and their interaction effects on all physiological indicators. Where significant main or interaction effects were found, the Least Significant Difference (LSD) test was applied for *post-hoc* multiple comparisons.

The landscape types compared in this study were predefined in the experimental design with clear theoretical hypotheses, rather than large-scale exploratory screening. The LSD test was adopted to improve the sensitivity of statistical tests and identify potential differences among various landscape stimuli. Normality and other basic assumption tests were performed before ANOVA. The results demonstrated that the data met the application requirements of this statistical method. All experimental results are presented as Mean ± SD, and the significance level was set at *p* < 0.05.

Although unequal variances existed among some landscape groups, the sample size was fully balanced for the audio group and silent group (*n* = 22). According to statistical theories, two-way ANOVA has strong robustness against mild and moderate heterogeneity of variance under balanced designs. Thus, two-way ANOVA was deemed appropriate for statistical inference in this study.

Each participant viewed 13 landscape videos sequentially. Several control measures were implemented to address the non-independence of repeated observations. The Schulte Grid task was used before each video to standardize participants’ stress levels and eliminate carryover effects from previous trials. Each stimulus lasted only 30 s, and a 1-min rest was arranged between trials to relieve visual fatigue and sequence interference. With these controls, the observations for different landscape scenes approximately satisfied the independence assumption. Two-way ANOVA was therefore used for subsequent statistical tests.

### 3.1 Analysis of stress recovery based on skin conductance level

The results of two-way ANOVA for skin conductance level (SCL) showed that the main effect of auditory environment was not significant (*p* = 0.091). The main effect of visual environment reached significance (*p* = 0.009), and the interaction between auditory and visual environments was also significant (*p* = 0.002) (see Table 7).

**Table 7:** Two-factor analysis of variance for skin conductance levels.

Source	Type III sum of squares	Degrees of freedom	Mean square	*F*	Significance (p)
Auditory environment	1.744	1	1.744	2.878	0.091
Visual environment	16.448	12	1.371	2.262	0.009*
Auditory * visual	18.978	12	1.582	2.610	0.002*

*Indicates *p* < 0.05, reaching the significance level.

The Least Significant Difference (LSD) *post-hoc* test was conducted for pairwise comparisons among different landscape groups (see Table 8). Significant differences were found as follows: Group A1 differed significantly from Groups A2, B1, B2, B3, C1, D1 and D3; Group A3 had significant differences with Groups B2, B3, C1 and D3. In addition, Groups B1, B2, B3, and C1 all showed significant differences from Groups C3 and C4. Significant differences also existed between Groups C3, C4 and Groups D1, D3.

**Table 8:** LSD *post-hoc* test for skin conductance level (*p* < 0.05).

Environmental factor (I)	Environmental factor(J)	Mean difference (I-J)	standard error	Significance (p)
A1	A2	0.3942^*^	0.1891	0.038^*^
	B1	0.4801^*^	0.2015	0.018^*^
	B2	0.5138^*^	0.1917	0.008^*^
	B3	0.5176^*^	0.1891	0.006^*^
	C1	0.5390^*^	0.1978	0.007^*^
	D1	0.5187^*^	0.1946	0.008^*^
	D2	0.2754	0.1917	0.152
	D3	0.6226^*^	0.2015	0.002^*^
A3	B2	0.3839^*^	0.1950	0.050^*^
	B3	0.3877^*^	0.1925	0.045^*^
	C1	0.4090^*^	0.2010	0.043^*^
	D3	0.4926^*^	0.2046	0.016^*^
B1	C3	−0.4183^*^	0.2015	0.038^*^
	C4	−0.4328^*^	0.1987	0.030^*^
B2	C3	−0.4520^*^	0.1917	0.019^*^
	C4	−0.4666^*^	0.1888	0.014^*^
B3	C3	−0.4558^*^	0.1891	0.016^*^
	C4	−0.4704^*^	0.1862	0.012^*^
C1	C3	−0.4772^*^	0.1978	0.016^*^
	C4	−0.4917^*^	0.1950	0.012^*^
C3	D1	0.4569^*^	0.1946	0.019^*^
	D3	0.5608^*^	0.2015	0.006^*^
C4	D1	0.4714^*^	0.1917	0.014^*^
	D3	0.5753^*^	0.1987	0.004^*^

*Indicates *p* < 0.05, reaching the significance level.

### 3.2 Analysis of stress recovery based on heart rate variability

The two-way ANOVA results for heart rate variability (HRV) are presented in Table 9. The main effect of auditory environment was not significant (*p* = 0.125). By contrast, the main effect of visual environment and the interaction effect between auditory and visual environments were both significant, with all *p* values less than 0.01.

**Table 9:** Two-factor analysis of variance for heart rate variability level.

Source	Type III sum of squares	Degrees of freedom	Mean square	*F*	Significance (p)
Auditory environment	56.648	1	56.648	2.367	0.125
Visual environment	905.889	12	75.491	3.155	0.000*
Auditory * visual	920.105	12	76.675	3.204	0.000*

*Indicates *p* < 0.05, reaching the significance level.

The LSD *post-hoc* test was performed to identify pairwise differences across groups (see Table 10). Significant differences were observed between Group A1 and Groups A3, B1, and C1. Group A2 also differed significantly from Groups A3, B1, and C1. Significant differences existed between Group A3 and Groups B2, D2. Group B1 showed significant disparities with Groups B2, B3, C2, C3, C4, D1, D2, and D3. A significant difference was found between Group B2 and Group C1. Moreover, Group C1 had significant differences relative to Groups C3, C4 and D2.

**Table 10:** LSD *post-hoc* test for heart rate variability level (*p* < 0.05).

Environmental factor (I)	Environmental factor(J)	Mean difference (I-J)	Standard error	Significance (p)
A1	A3	−2.4568*	1.0952	0.025*
	B1	−4.0332*	1.0807	0.000*
	C1	−2.7563*	1.0952	0.012*
A2	A3	−2.1966*	1.1081	0.048*
	B1	−3.7730*	1.0938	0.001*
	C1	−2.4961*	1.1081	0.025*
A3	B2	2.6686*	1.1081	0.016*
	D2	2.9399*	1.1377	0.010*
B1	B2	4.2450*	1.0938	0.000*
	B3	3.0099*	1.0807	0.006*
	C2	3.3948*	1.1081	0.002*
	C3	3.7685*	1.1081	0.001*
	C4	3.6391*	1.1081	0.001*
	D1	2.6770*	1.0807	0.014*
	D2	4.5163*	1.1238	0.000*
	D3	3.0754*	1.1081	0.006*
B2	C1	−2.9681*	1.1081	0.008*
C1	C3	2.4916*	1.1222	0.027*
	C4	2.3621*	1.1222	0.036*
	D2	3.2394*	1.1377	0.005*

*Indicates *p* < 0.05, reaching the significance level.

### 3.3 Analysis of stress recovery based on electroencephalogram signals

The two-way ANOVA results of EEG signals are shown in Table 11. The main effect of auditory environment was not significant (*p* = 0.585). The main effect of visual environment was significant (*p* = 0.03), and the interaction effect between auditory and visual environments was also significant (*p* = 0.002).

**Table 11:** Two-factor analysis of variance for EEG signals.

Source	Type III sum of squares	Degrees of freedom	Mean square	*F*	Significance (p)
Auditory environment	39.938	1	39.938	0.299	0.585
Visual environment	3068.216	12	255.685	1.916	0.030*
Auditory * visual	3349.382	12	279.115	2.091	0.016*

*Indicates *p* < 0.05, reaching the significance level.

The LSD *post-hoc* test was adopted to compare significant differences among groups (see Table 12). The results indicated that Group C3 had statistically significant differences with all other groups. In addition, a significant difference existed between Group B1 and Group B2.

**Table 12:** LSD *post-hoc* test for EEG signals (*p* < 0.05).

Environmental factor (I)	Environmental factor(J)	Mean difference (I-J)	Standard error	Significance (p)
A1	C3	−6.1430*	2.1644	0.005*
A2	C3	−7.5366*	2.1644	0.001*
A3	C3	−5.6759*	2.1644	0.009*
B1	B2	4.7250*	2.1644	0.029*
	C3	−4.4577*	2.1453	0.038*
B2	C3	−9.1827*	2.1644	0.000*
B3	C3	−7.7292*	2.1644	0.000*
C1	C3	−5.3669*	2.1644	0.013*
C2	C4	−6.9660*	2.1453	0.001*
C3	C4	6.2375*	2.1644	0.004*
	D1	6.9336*	2.1644	0.001*
	D2	5.3588*	2.1644	0.014*
	D3	6.5660*	2.1644	0.003*

*Indicates *p* < 0.05, reaching the significance level.

### 3.4 Analysis of stress recovery based on eye movement indicators

Two-way ANOVA was conducted separately for three eye movement indicators: mean pupil diameter, mean blink frequency and mean saccade frequency. The results are summarized in Tables 13–15.

**Table 13:** Two-factor analysis of variance for average pupil diameter.

Source	Type III sum of squares	Degrees of freedom	Mean square	*F*	Significance (p)
Auditory environment	8.277	1	8.277	15.732	0.000*
Visual environment	6.060	12	0.505	0.960	0.486
Auditory * visual	2.276	12	0.190	0.361	0.976

*Indicates *p* < 0.05, reaching the significance level.

**Table 14:** Two-factor analysis of variance for average blink frequency.

Source	Type III sum of squares	Degrees of freedom	Mean square	*F*	Significance (p)
Auditory environment	6.477	1	6.477	50.264	0.000*
Visual environment	1.989	12	0.166	1.286	0.221
Auditory * visual	1.819	12	0.152	1.176	0.296

*Indicates *p* < 0.05, reaching the significance level.

**Table 15:** Two-factor analysis of variance for average saccade frequency.

Source	Type III sum of squares	Degrees of freedom	Mean square	F	Significance (p)
Auditory environment	80.216	1	80.216	16.326	0.000*
Visual environment	48.282	12	4.024	0.819	0.631
Auditory * visual	114.510	12	9.543	1.942	0.027*

*Indicates *p* < 0.05, reaching the significance level.

The main effect of auditory environment was significant for all three indicators, with all *p* values less than 0.01. No significant main effect of visual environment was detected for any indicator. The interaction effect between auditory and visual environments was only significant for mean saccade frequency (*p* = 0.027), while the interaction terms of the other two indicators did not reach statistical significance.

**Figure 4:**
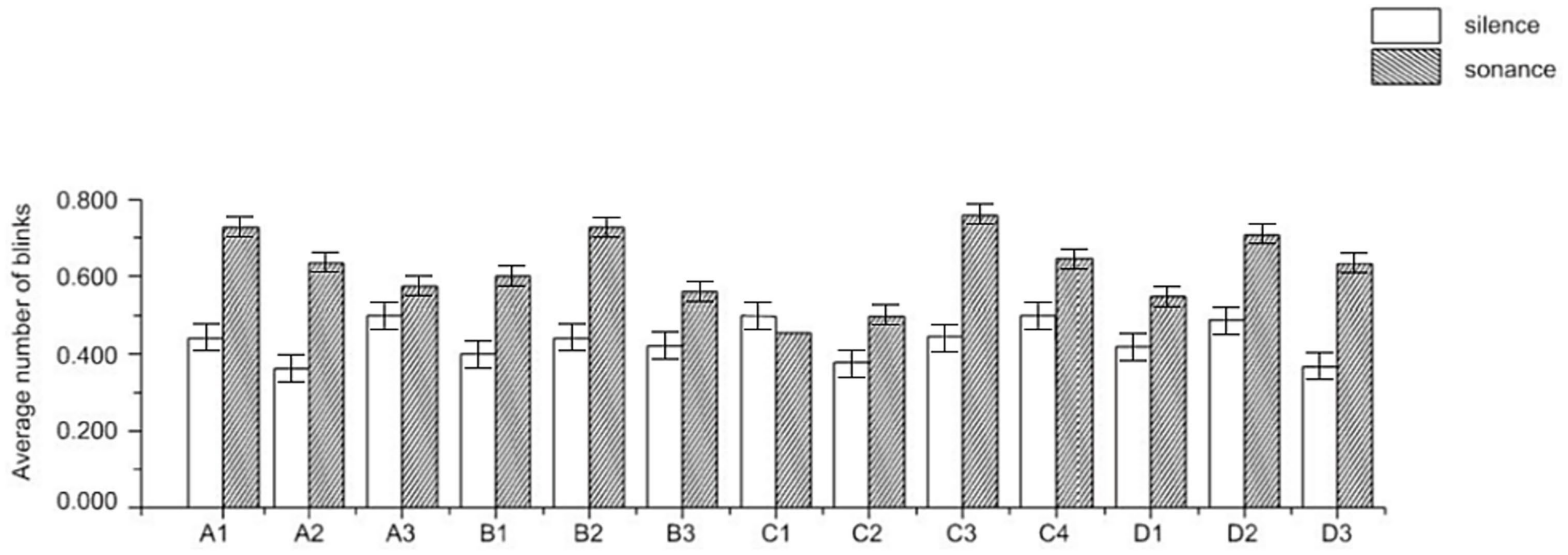
Statistical data of average pupil diameter.

**Figure 5:**
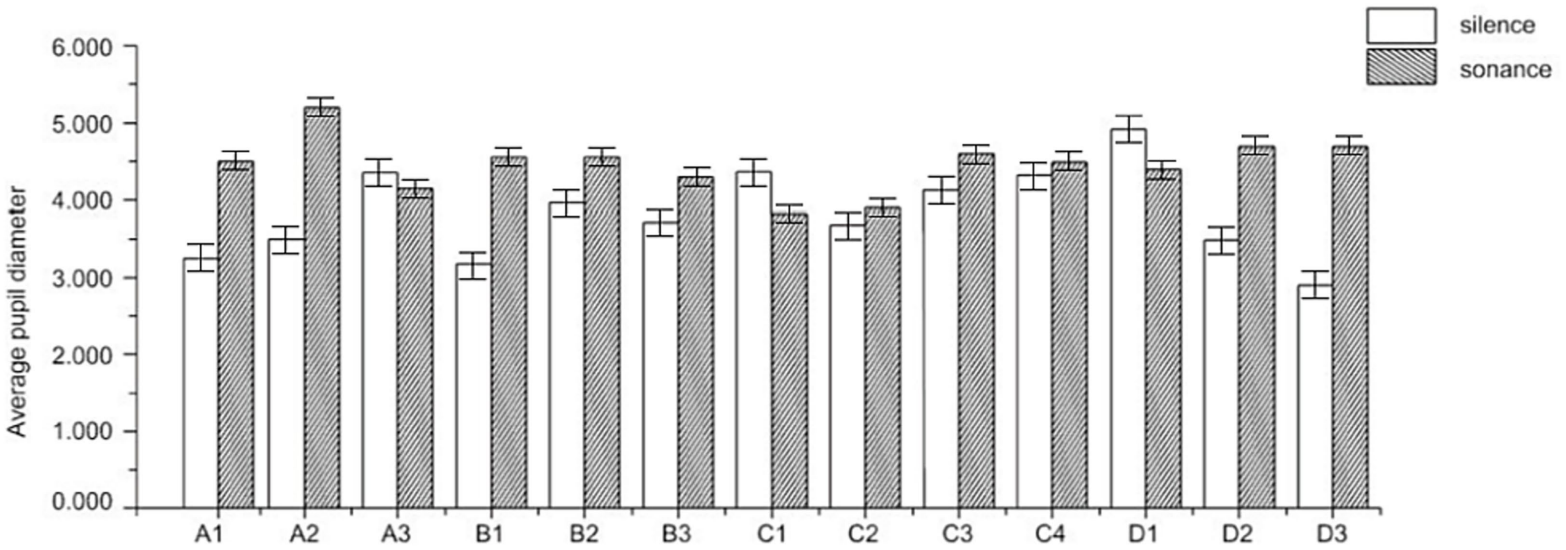
Statistical data of average blink frequency.

**Figure 6:**
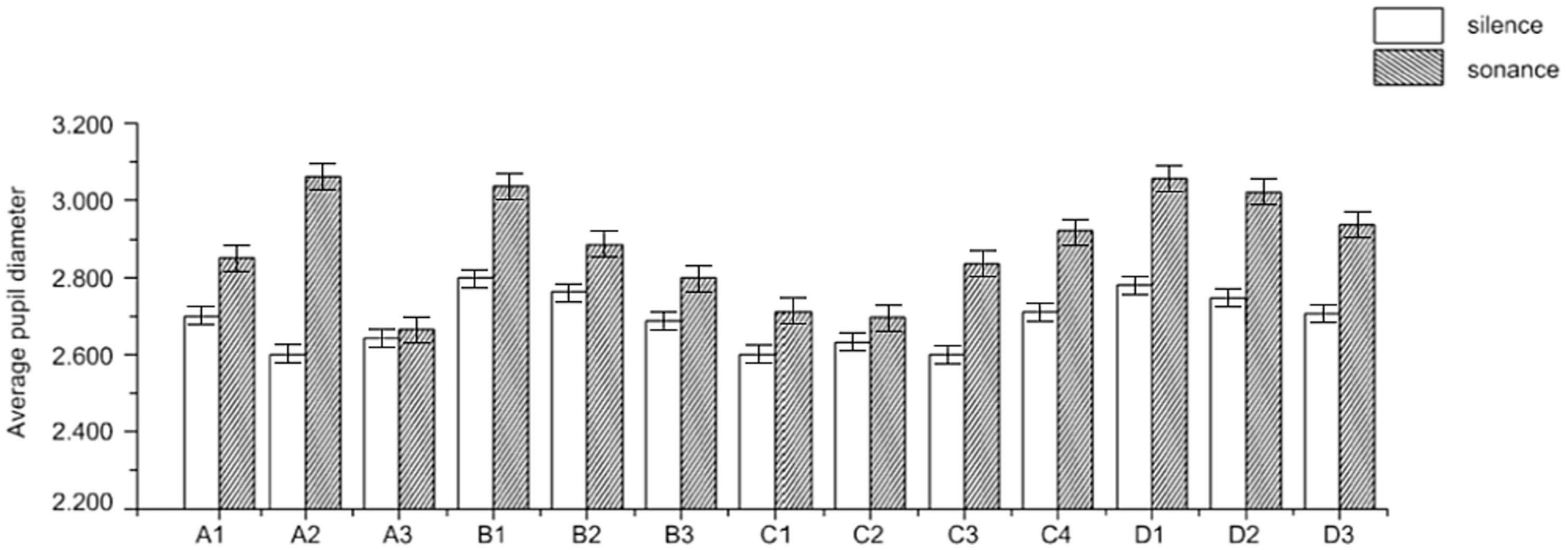
Statistical data of average saccade frequency.

As illustrated in Figures 4–6, most eye movement indicators of the audio-present group were higher than those of the silent group. Specifically, the mean blink frequency rose under audio conditions at sites A1, B2, C3, and D2. The mean saccade frequency increased for sites A2, B1, D2, and D3 with sound, yet decreased at A3, C1, and D1.

In terms of landscape categories: within Group A, sites A1 and A2 showed generally elevated eye movement indicators in the audio group, while the variation of A3 was mild. For Group B, B3 maintained relatively low eye movement values under both audio and silent conditions. In Group C, C3, and C4 yielded overall higher eye movement indicators with obvious discrepancies between the two auditory treatments, whereas C1 presented reduced average saccade frequency when sound was available. As for Group D, D2, and D3 had higher eye movement indicators in the audio group, and D1 showed negligible changes.

**Figure 7:**
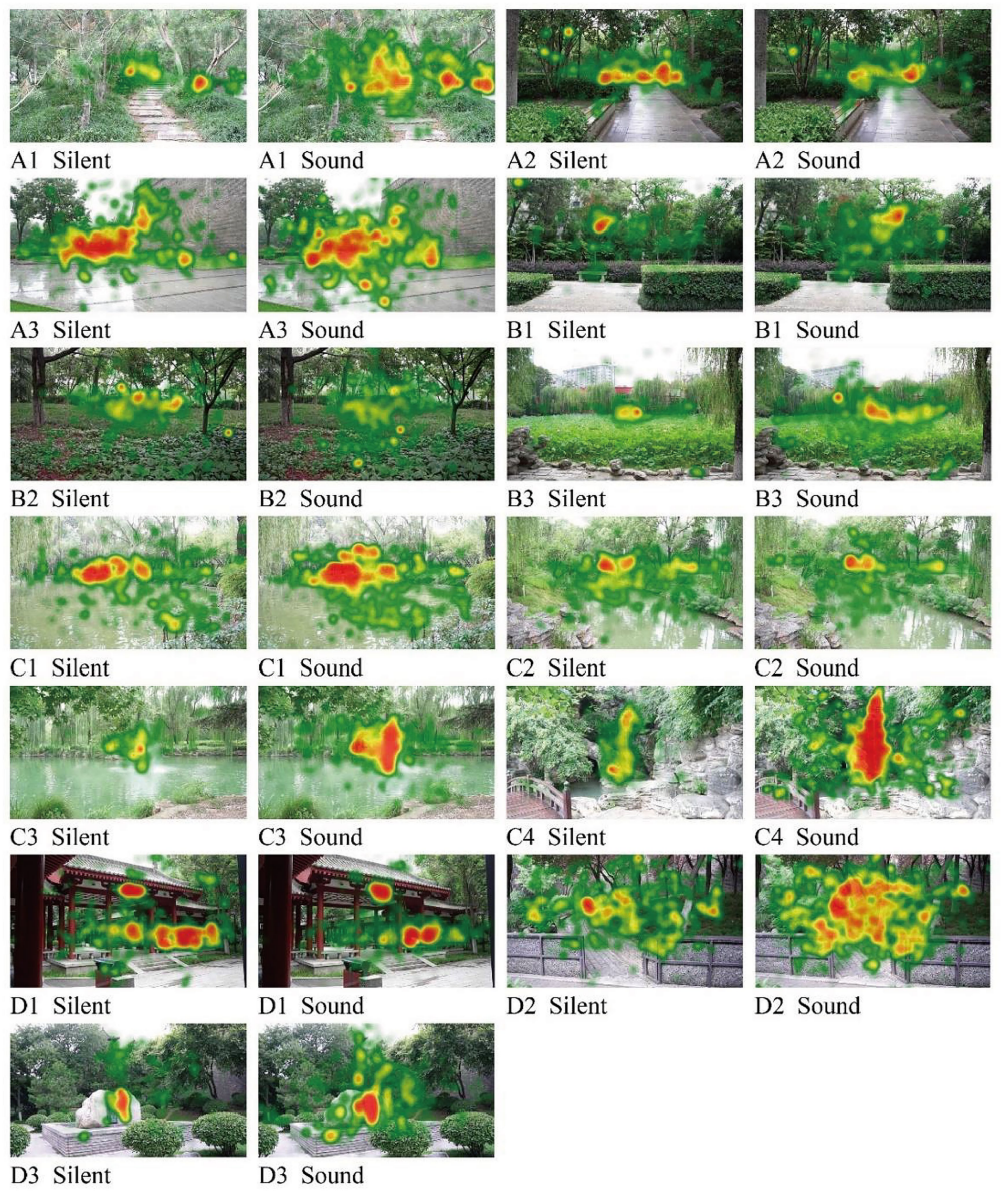
Eye-tracking heatmap.

Aggregated visual heatmaps of all participants (Figure 7) revealed that participants’ attention mainly converged on the visual center, the end of paths, as well as vegetation and water features. Compared with the silent group, the audio group exhibited more concentrated fixation distribution and paid greater attention to water landscapes.

Shapiro–Wilk normality tests were performed on HRV, SCL, EEG and eye-tracking data stratified by auditory group and landscape scene. Moderate to severe non-normal distribution was detected in a small number of subgroups, while most landscape subsets satisfied the normality assumption. Such skewed distributions are common in physiological experiments concerning environmental perception and stress recovery, primarily resulting from individual physiological differences among older adult participants and inherent characteristics of bio-signals. Given the perfectly balanced sample sizes of the audio and silent groups, two-way ANOVA is robust to moderate non-normality, which guarantees the reliability of the statistical results.

Two-way ANOVA was applied to test the main effects of auditory environments, visual environments and their interaction effects on all indicators. Where significant main or interaction effects emerged, the Least Significant Difference (LSD) test was adopted for *post-hoc* multiple comparisons. The landscape types to be compared were predefined in the experimental design with clear theoretical hypotheses rather than exploratory screening across numerous variables. The LSD test was therefore selected to enhance statistical sensitivity and identify subtle differences among landscape stimuli. The findings provide references for optimizing the stress-reduction benefits of rehabilitative landscapes for older adults in urban parks, and the core conclusions are summarized as follows:

First, visual environments exerted significant main effects and interacted significantly with auditory environments for SCL, HRV and EEG signals. This demonstrates that visual settings are a decisive factor in older adults’ stress recovery. The restorative benefits of sound do not function independently but are modulated by visual landscape types. The effectiveness of auditory stimuli is conditional, and the combined audio-visual interaction delivers stronger stress relief. Meanwhile, Group B (moderate plant diversity) and Group C (water features) yielded higher physiological recovery rates than other groups, indicating that abundant natural vegetation and water landscapes produce superior stress recovery effects.

Second, eye movement data revealed that auditory input reshapes visual information processing. Sound may raise visual load through multi-sensory competition and trigger psychological stress. Nevertheless, open space A3 and static water scene C1 showed stable eye-tracking indicators with minimal sound interference, reflecting strong anti-disturbance capacity.

Third, visual heatmap analysis proved that ambient sound strengthens participants’ fixation on water bodies and structural landscape elements and stabilizes visual attention. However, intensified concentration may lead to information overload and excessive consumption of cognitive resources. This partly explains why the combination of sound and complex dynamic water features (fountains, waterfalls) elevates cognitive load and impairs stress recovery instead.

Fourth, LSD *post-hoc* comparisons quantified the distinct effects of various environment combinations. The results confirmed that integrated audio-visual stimuli generate better stress recovery outcomes than visual-only stimuli for older adults.

Overall, the optimal restorative environment combinations feature open spaces with moderate visual complexity. A representative example is open space A3 matched with medium plant richness B2. In addition, static water scene C1 exhibited reduced saccade frequency under audio conditions, serving as an effective stress buffer. For dynamic water features and artificial structures, sound design should be carefully regulated to avoid audio-visual overload that weakens the therapeutic effects of age-friendly rehabilitative landscapes.

## 4 Discussion

This study systematically explored the mechanism through which rehabilitative landscapes relieve stress among older adults, and verified its effects via questionnaire surveys and empirical tests. The results demonstrate that rehabilitative landscapes designed based on older adults’ landscape perception can significantly facilitate their stress recovery and exert positive impacts on their mental state and emotional well-being.

The experimental data reveal that landscapes with high landsense evaluation scores trigger more prominent physiological recovery responses, which aligns with the core viewpoint of landsenses theory emphasizing the importance of environmental perception. This study also confirms the critical role of environmental perception in people’s psychological experience.

Furthermore, different dimensions of landscape perception exert significantly distinct effects on stress recovery. Perceptual factors including safety, comfort and accessibility produce the most pronounced benefits for older adults’ stress relief, indicating functional guarantees and psychological security are fundamental prerequisites for restorative experiences. Additionally, perceptions of natural elements and social interaction also contribute positively to stress recovery, which highlights the necessity of integrating ecological components and social communication spaces into rehabilitative landscape design.

Empirically, this study clarifies how audio-visual environments of various rehabilitative landscapes affect stress recovery in older adults, supplements evidence on multi-sensory experiences of the elderly within restorative environment research, and further unpacks the internal pathways linking environmental perception to stress recovery. These findings not only enrich the theoretical framework of rehabilitative and restorative environment research, but also provide practically valuable references for the planning, design and renewal of urban rehabilitative landscapes amid population aging.

### 4.1 Interpretation of core findings

This study confirms that visual landscape characteristics play a dominant role in facilitating older adults’ stress recovery, while the restorative effects of auditory stimuli are contingent on specific visual contexts. This finding indicates that stress recovery in therapeutic landscapes is not driven by a single sensory channel, but arises from the interaction between visual and auditory environmental information.

Among all landscape elements examined, vegetation and water bodies deliver stronger restorative potential than built structures. In particular, environments with moderate visual complexity elicit more stable recovery responses. These results support the propositions of landsenses theory: individuals respond to environmental features through integrated multi-sensory experiences, which further shape their psychological and physiological recovery status.

### 4.2 Comparison with prior research

The positive effects of natural landscape elements observed in this research are generally consistent with previous studies grounded in Stress Reduction Theory and Attention Restoration Theory. Early literature has proven that vegetation and water features effectively mitigate psychological stress and improve emotional health.

However, unlike most prior studies that focus merely on visual stimuli, this research highlights the vital role of audio-visual interaction. The results show that auditory sounds do not always amplify restorative effects; their efficacy varies depending on visual landscape features. For instance, dynamic water scenes show stronger responses to auditory input, whereas static water bodies yield stable physiological and eye-tracking outcomes. These discoveries demonstrate that the therapeutic potential of rehabilitative landscapes depends not only on individual environmental components, but also on the interactive modes of diverse sensory cues.

### 4.3 Implications for therapeutic landscape design

The outcomes deliver practical design guidelines for therapeutic landscapes serving older adults. First, natural elements such as vegetation and water should be prioritized due to their positive correlation with stress alleviation. Second, landscapes with moderate visual complexity outperform overly simplified or highly intricate environments. Designers therefore need to strike a reasonable balance between visual richness and environmental legibility.

Moreover, auditory elements should be selectively incorporated rather than added arbitrarily. Although natural sounds can enhance restorative experiences in certain contexts, combining excessive auditory stimulation with dynamic visual scenes may weaken therapeutic benefits. Accordingly, therapeutic landscape design requires coordinated visual and auditory layout to create balanced multi-sensory experiences for older adults.

### 4.4 Limitations and future research directions

Several limitations of this study need to be acknowledged.

First, constraints exist in the statistical approach. Each participant viewed a sequence of video clips, generating repeated-measure data. Conventional two-way ANOVA cannot fully eliminate the non-independence of observations. Restricted by objective experimental conditions, this study did not re-analyze data using mixed-design ANOVA or linear mixed-effects models. Future relevant research will adopt statistical models suited for repeated measurement structures to improve result accuracy.

Second, effect sizes and confidence intervals were not reported in this paper; only *p*-values were used to judge significant differences. Constrained by original calculation files and equipment conditions, corrected *post-hoc* tests and effect size computation cannot be rerun at this stage. Follow-up experiments will strictly adopt correction methods including Tukey and Bonferroni adjustments, and supplement effect sizes and confidence intervals to perfect the statistical inference system.

Third, landscape videos were adopted as experimental stimuli instead of real-site environments. While video stimuli enable standardized experimental conditions, they fail to fully replicate the multi-sensory and immersive characteristics of authentic therapeutic landscapes. Subsequent research may adopt virtual reality (VR) or field experiments to elevate ecological validity and simulate real environmental experiences more authentically.

Fourth, the sample size is relatively small and all participants were older adults from a single geographic area. The generalizability of the findings should thus be interpreted cautiously. Future studies will recruit larger, more diverse samples covering multiple cities, cultural backgrounds and age groups to further validate the conclusions.

Fifth, this study only focuses on short-term acute stress recovery responses. Further research can explore the long-term benefits of repeated exposure to rehabilitative landscapes, and investigate how landscape perception shapes long-term health and well-being.

Finally, although the 30-s stimulus duration reliably captures older adults’ immediate recovery reactions under consistent experimental conditions, longer stimulus windows may generate more stable estimates for frequency-domain HRV parameters. Future work can compare measurement outcomes of stress recovery across different stimulus durations.

## 5 Optimization strategies and methods for rehabilitative landscape design

Based on questionnaire surveys on older adults’ subjective demands and experimental results of physiological indicators, EEG and eye movement tracking, this study analyzed how combinations of diverse landscape elements and audio-visual environments affect stress recovery among the elderly. The results reveal that natural landscapes, coordinated multi-sensory stimulation and rational spatial layout can significantly improve restorative experiences. Drawing on the above experimental findings, this paper puts forward targeted design strategies for age-friendly rehabilitative landscapes, so as to support the renewal of urban parks and community green spaces.

### 5.1 Strengthen the restorative value of natural visual settings

Experimental results prove that visual environments exert a significant influence on older adults’ stress recovery (Bi and Wu, 2024). Compared with highly artificial landscapes, natural scenes featuring diverse vegetation, harmonious water features and moderate openness produce stronger physiological restorative effects. Eye movement data further indicate that older adults fixate longer and concentrate more on natural elements including plants and water bodies.

Accordingly, age-friendly rehabilitative landscape design should prioritize abundant vegetation layouts, integrated water scenery and continuous natural elements. Moderately layered plant communities and open, comfortable activity zones should be constructed to boost the landscape’s therapeutic potential (Li et al., 2022). Meanwhile, sufficient safety and accessibility must be guaranteed to create a stable and pleasant outdoor space for older adults.

### 5.2 Focus on harmonious matching of visual and auditory environments

Physiological experiments demonstrate that auditory environments alone do not generate stable main effects, yet significant interaction exists between sound and visual landscapes. It suggests the stress-relieving benefits of acoustic environments are highly context-dependent. In addition, eye-tracking data show that extra auditory stimuli may raise visual information processing load under certain landscape conditions, weakening restorative outcomes.

Therefore, rehabilitative landscape design should pursue coordination between sight and sound rather than simply adding acoustic elements. Natural sounds such as birdsong and flowing water can be appropriately arranged within vegetated areas and waterscapes to enrich environmental experience. For busy zones with dense artificial facilities, ambient noise should be controlled to prevent complex sound sources from imposing extra cognitive burden on older adults.

### 5.3 Emphasize integrated multi-sensory landscape experience

Combining experimental results with existing theories of restorative environments, this study confirms that the therapeutic effects of rehabilitative landscapes stem from integrated multi-source environmental signals, instead of merely visual or auditory input. Both physiological signals and eye movement behaviors verify that consistent environmental perception improves older adults’ restorative experience.

Age-friendly rehabilitative landscape design should value the synergy among vision, hearing and overall environmental sensation. Integrated construction of vegetation, water bodies, natural soundscapes and comfortable microclimates helps create inclusive, restorative multi-sensory landscape spaces.

It should be noted that the above design inspirations are derived mainly from physiological and eye-tracking data in this study. Broader strategies for landscape planning, facility arrangement and park operation management require further verification in future research.

## 6 Conclusion

Combining human factors engineering, multimodal physiological monitoring and questionnaire surveys based on the Kano model, this study took typical urban parks in Xi’an as research objects to systematically explore the mechanisms through which various age-friendly rehabilitative landscape elements relieve older adults’ stress under visual-only and audio-visual conditions. By integrating objective physiological measurements and subjective demand analysis, this paper clarified the internal correlations between landscape configuration, sensory coordination and stress recovery of the elderly.

The results show that landscape elements differ significantly in their restorative effects. Landscapes with moderate plant diversity and static water features deliver more stable and prominent stress relief. Compared with visual stimuli alone, integrated audio-visual environments effectively strengthen stress recovery and guide visual attention toward core landscape elements. Furthermore, open spaces possess strong anti-interference capacity under multi-sensory stimulation, reducing cognitive load and maintaining stable perceptual states.

This study identifies key landscape elements and their multi-sensory coordination mechanisms conducive to older adults’ stress recovery. It provides scientific evidence for optimizing age-friendly rehabilitative landscapes in urban parks and community green spaces, and offers references for research on healthy aging environments from a multi-sensory synergy perspective.

## Data Availability

The original contributions presented in the study are included in the article/supplementary material, further inquiries can be directed to the corresponding author.
